# Factors Associated With Failure of Synthesis in the Treatment of Proximal Femur Fractures With Cephalomedullary Nails

**DOI:** 10.7759/cureus.61363

**Published:** 2024-05-30

**Authors:** Luis Henrique Longo, Heloisa Zimmermann Faggion, Matheus Costa Sartor, Matheus U Senna Klipp, Paulo Henrique Vogt, Alberto Daniel Navarro Vergara, Weverley R Valenza

**Affiliations:** 1 Department of Orthopedics and Traumatology, Hospital do Trabalhador, Curitiba, BRA; 2 Department of Pediatrics, Child Orthopedics and Traumatology Unit, Hospital de Trauma Manuel Giagni, Asunción, PRY

**Keywords:** nail failure, implant breakage, cut-out, cephalomedullary nail, pertrochanteric fracture

## Abstract

Introduction: Proximal femur fractures are common in older patients and typically require surgical treatment, with cephalomedullary nails being the gold standard device for this approach. This study aimed to identify the factors associated with the failure of cephalomedullary nailing.

Materials and methods: We retrospectively evaluated 380 patients treated with a cephalomedullary nail between August 2021 and August 2022 in a trauma referral center in Brazil. A total of 221 (58.1%) patients were included in the study after applying specific eligibility criteria. Data were collected and rates were determined by reviewing patients' medical records and radiographs.

Results: Of 221 patients, 14 (6.3%) had nail failures A significant association was found between post-fixation cervico-diaphyseal angle and the occurrence of nail failure (p<0.001). Furthermore, calcar-referenced tip-apex distance (CalTAD) and tip-apex distance (TAD) values were higher in cases with nail failure than in those without nail failure. Cutoff points were established for TAD and CalTAD to measure the correspondence with nail failures.

Conclusion: The present study supports previous evidence that varus reduction potentially causes collapse and nail failure in pertrochanteric fractures treated with cephalomedullary nailing and that high TAD and CalTAD values contribute to the incidence of cut-out.

## Introduction

Fractures of the proximal femur typically affect older patients, with an increasing incidence as the population ages [1]. These fractures are challenging not only for trauma surgeons but also for society as they are associated with high patient morbidity and mortality and high treatment costs [2]. Pertrochanteric fractures affect patients slightly older than those affected by femoral neck fractures. They are more common in females over 70 years of age [3]. Surgery is the gold standard treatment, adopted in the vast majority of cases. In recent years, the conservative approach has been less commonly used, being reserved for specific cases with multiple comorbidities that contraindicate surgery [1-3].

Cephalomedullary nails have gained popularity in the surgical treatment of pertrochanteric fractures due to reduced operative time and intraoperative blood loss, proving to be a stable implant that allows for early postoperative mobilization. Furthermore, as an intramedullary implant, it has improved the safety of approaches to fractures involving the lateral wall and lesser trochanter as well as to reverse oblique fractures (AO 31-A2 and AO 31-A3, respectively) [4].

Despite the advantages associated with cephalomedullary nailing, failures still occur. Failure may occur due to the severity or natural instability of the fracture, poor reduction quality, or collapse of the implant used. Nail failure is a serious complication, as it requires surgical reintervention, exposing a geriatric population to prolonged hospitalizations in which 30-day mortality rates reach 8% [5].

The most common cause of failure of fixation is cut-out, but other major factors include cut-through, varus reduction, and hardware breakage. Predictors of failure have been extensively investigated, especially for dynamic (sliding) hip screw (DHS) fixation, in studies conducted by Baumgaertner et al. [6]. However, there are significant biomechanical differences between DHS and cephalomedullary nails: the variation in the location of contact of the screw with the femoral axis and the possibility of lag screw locking in these nails. Measurement of the position of the lag screw using techniques such as the tip-apex distance (TAD) [6] and later the calcar-referenced tip-apex distance (CalTAD) [7] has contributed to both the debate about the optimal position of the lag screw and intraoperative management to prevent failures, especially cut-out [8].

The present study aimed to evaluate the factors associated with the failure of cephalomedullary nailing of pertrochanteric fractures.

## Materials and methods

We retrospectively evaluated 380 patients with pertrochanteric fractures (AO 31-A1, 31-A2, 31-A3) who were treated with a cephalomedullary nail (short or long) between August 2021 and August 2022 in a tertiary trauma referral center.

Data were collected by reviewing patients' medical records and radiographs. Exclusions included 48 (12.6%) patients who died preoperatively or postoperatively, 15 (3.9%) patients treated conservatively or treated with other types of implants, such as DHS or arthroplasty, and 96 (25.2%) patients who did not complete at least six months of postoperative outpatient follow-up or had incomplete medical records and missing information. Therefore, 221 (58.1%) patients treated with a cephalomedullary nail during the study period with a minimum outpatient follow-up of six months postoperatively were included in the study.

The surgical procedures were performed by more than one surgeon, including experienced orthopedists and traumatologists, as well as residents at different levels of graduation. The implants used during the study period were cephalomedullary gamma nails, models Fênix® (Biomecânica Industria Ltda, SP, Brazil) and Haste Fêmur III® (Hexagon Industria Ltda, SP, Brazil).

The technique adopted consisted of positioning the anesthetized patient on a traction table. Functional indirect closed reduction was performed in 217 (98.2%) patients, whereas direct open reduction was performed in only four (1.8%) patients.

Early postoperative physical therapy was indicated, and patients were allowed to weight bearing as tolerated on the operated leg and to ambulate with the aid of a walker. Active and passive mobilization of the hip was also recommended, without limiting the range of motion.

The data collected for analysis were sex, age, post-fixation cervico-diaphyseal angle (<125° varus; >125-145° neutral; >145° valgus), post-reduction medial cortex support (positive, neutral, or negative reduction), presence of complementary osteosynthesis, TAD (mm), CalTAD (mm), lateral screw breach (mm), nail failure (yes or no), and mode of nail failure.

The data were entered into an Excel® spreadsheet and analyzed using IBM SPSS Statistics for Windows, Version 28.0 (Released 2021; IBM Corp., Armonk, New York, United States). Quantitative variables were expressed as mean (SD) or median (minimum and maximum). Categorical variables were expressed as absolute numbers and percentages. Factors associated with the occurrence of nail failure were analyzed using Student's t-test for independent samples (quantitative variables) and Fisher's exact test or chi-squared test (categorical variables). Receiver operating characteristic (ROC) curves were adjusted to define cutoff points for TAD (mm) and CalTAD (mm) considering nail failure. The cutoff points were determined by the Youden index. A two-sided p-value <0.05 was considered statistically significant.

Consent was obtained or waived by all participants in this study. The study was approved by the Research Ethics Committee of Hospital do Trabalhador/SES, Paraná, Brazil, under number CAAE: 78817124.9.0000.5225.

## Results

Data from 221 patients with pertrochanteric fractures who were surgically treated with cephalomedullary nails (short or long) at our institution between August 2021 and August 2022 were analyzed.

Table 1 shows the descriptive statistics for each variable evaluated in the study.

**Table 1:** Demographic and clinical variables of study participants (n=221). CalTAD: calcar-referenced tip-apex distance; TAD: tip-apex distance

Variable	Statistics
Age (years), mean±SD	75.0±15.5
Median (min-max)	80.0 (20-101)
Sex, n (%)	
Female	151 (68.3)
Male	70 (31.7)
Cervico-diaphyseal angle, n (%)	
Neutral	164 (74.2)
Valgus	10 (4.5)
Varus	47 (21.3)
Reduction, n (%)	
Negative	36 (16.3)
Neutral	99 (44.8)
Positive	86 (38.9)
Complementary osteosynthesis, n (%)	
No	217 (98.2)
Yes	4 (1.8)
TAD (mm), mean±SD	20.8±6.5
Median (min-max)	19.6 (8.5-46)
CalTAD (mm), mean±SD	26.1±6.2
Median (min-max)	25.8 (6.5-51)
Lateral screw breach (mm), mean±SD	10.0±4.6
Median (min-max)	9.8 (0-23.3)

Of 221 patients, 207 (93.7%) had no nail failures and 14 (6.3%) had nail failures (Table 2).

**Table 2:** Variables related to nail failure and TAD and CalTAD indices. *Cutoff point suggested by the ROC curve. CalTAD: calcar-referenced tip-apex distance; TAD: tip-apex distance; ROC: receiver operating characteristic

Variable	n	%
Nail failure		
No	207	93.7
Yes	14	6.3
Mode of nail failure		
Implant breakage	5	35.7
Cut-out	4	28.6
Cut-through	3	21.4
Varus collapse	2	14.3
TAD (mm)*		
≤20	112	50.7
≥20	109	49.3
CalTAD (mm)*		
≤27	128	57.9
>27	93	42.1

**Figure 1:**
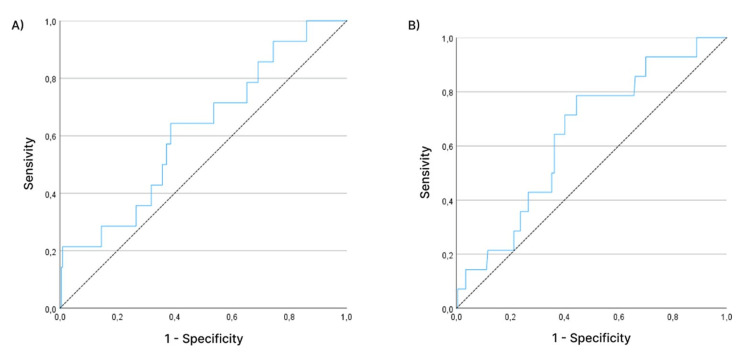
(A) TAD and (B) CalTAD ROC curves. CalTAD: calcar-referenced tip-apex distance; TAD: tip-apex distance; ROC: receiver operating characteristic

Figure 1 shows the ROC curves adjusted to define the cutoff points for TAD (mm) and CalTAD (mm) associated with nail failure.

For TAD, the area under the ROC curve was 0.64, with a trend towards statistical significance (p=0.080), that is, TAD tends to discriminate well between having and not having nail failure. We then determined a cutoff point for TAD, which, adjusted by the ROC curve, corresponded to 20 mm. Therefore, TAD <20 mm represented a protection factor, whereas TAD ≥20 mm tended to be related to failure. For this cutoff point, sensitivity was estimated at 78.6% and specificity at 55.6%.

For CalTAD, the area under the ROC curve was 0.62, without statistical significance (p=0.140). Even with this result, we determined a cutoff point for CalTAD. The optimal cutoff point, as adjusted by the ROC curve, corresponded to 27 mm, with CalTAD ≤27 mm being a protection factor and CalTAD >27 mm corresponding to failure. For this cutoff point, sensitivity was estimated at 64.3% and specificity at 61.4%.

For each of the variables, we tested the null hypothesis that there is no association between the variable and the occurrence of nail failure and the alternative hypothesis that there is an association. Table 3 shows the factors associated with the occurrence of nail failure.

**Table 3:** Association of factors with the occurrence of nail failure. *Student's t-test for independent samples (quantitative variables); Fisher's exact test or chi-squared test (categorical variables); significant at p<0.05. †Cutoff point suggested by the ROC curve. CalTAD: calcar-referenced tip-apex distance; TAD: tip-apex distance; ROC: receiver operating characteristic

Variable	Total	Nail failure		p*
		No	Yes	
Age (years), mean±SD	221	75.0±15.6	74.9±14.4	0.978
(min-max)		(20-99)	(37-101)	
Sex				1
Female	151	141 (93.4%)	10 (6.6%)	
Male	70	66 (94.3%)	4 (5.7%)	
Cervico-diaphyseal angle				-
Neutral	164	159 (97%)	5 (3%)	
Valgus	10	10 (100%)	0 (0%)	-
Varus	47	38 (80.9%)	9 (19.1%)	
Cervico-diaphyseal angle (cluster)				<0.001
Valgus/neutral	174	169 (97.1%)	5 (2.9%)	
Varus	47	38 (80.9%)	9 (19.1%)	
Reduction				0.159
Negative	36	32 (88.9%)	4 (11.1%)	
Neutral	99	96 (97%)	3 (3%)	
Positive	86	79 (91.9%)	7 (8.1%)	
Reduction (cluster)				0.253
Neutral/positive	185	175 (94.6%)	10 (5.4%)	
Negative	36	32 (88.9%)	4 (11.1%)	
Complementary osteosynthesis				1
No	217	203 (93.5%)	14 (6.5%)	
Yes	4	4 (100%)	0 (0%)	
Lateral screw breach (mm), mean±SD	221	9.9±4.5	11.4±6.5	0.240
(min-max)		(0.0-21.5)	(0.0-23.3)	
TAD (mm), mean±SD	221	20.6±6.4	23.9±7.0	0.061
(min-max)		(8.5-46)	(13.0-40.7)	
TAD (mm)†				0.028
	112	109 (97.3%)	3 (2.7%)	
≥20	109	98 (89.9%)	11 (10.1%)	
CalTAD (mm), mean±SD	221	25.9±6.1	29.2±7.3	0.048
(min-max)		(6.5-51.0)	(19.8-42.2)	
CalTAD (mm)†				0.098
≤27	128	123 (96.1%)	5 (3.9%)	
>27	93	84 (90.3%)	9 (9.7%)	

A significant association was found between post-fixation cervico-diaphyseal angle and the occurrence of nail failure (p<0.001). Of 174 cases with valgus/neutral reduction, only five (2.9%) had nail failure. However, of 47 cases with varus reduction, nine (19.1%) had nail failure.

The results also indicated a significant association between CalTAD and the occurrence of nail failures (p=0.048). Mean CalTAD values were higher for cases with nail failure than for those without nail failure.

Considering the CalTAD cutoff of 27 mm, there was a trend towards significance in the association of this variable with the occurrence of nail failure (p=0.098). Cases with CalTAD ≤27 mm were associated with a lower percentage of nail failure (n=5, 3.9%) than those with CalTAD >27 mm (n=9, 9.7%).

Considering the TAD cutoff of 20 mm, significant associations were found (p=0.028). Of 112 cases with TAD <20 mm, only three (2.7%) had nail failure. However, of 109 with TAD ≥20 mm, 11 (10.1%) had nail failure. A trend towards statistical significance was also observed for TAD (p=0.061), where mean TAD values were higher for cases with nail failure than for those without nail failure.

All other variables (factors) analyzed were not significantly associated with the occurrence of nail failure (Table 3).

## Discussion

Pertrochanteric fractures can be treated using multiple techniques, such as screw-plate fixation and cephalomedullary nailing [9]. These techniques were introduced into orthopedic practice in the late 1980s. In 1992, the development of long cephalomedullary nails expanded their indications especially to include unstable fractures, which further popularized the use of these nails, now including pertrochanteric fractures with subtrochanteric extension, subtrochanteric fractures, reverse oblique intertrochanteric fractures, and pathological fractures [10,11]. Compared with DHS fixation, cephalomedullary nailing is associated with reduced operative time and intraoperative blood loss, in addition to allowing for early ambulation even in unstable fractures [12-14].

With the increased use of nails, some failures began to be investigated. Cut-out is the most prevalent cause of failure reported in the literature. In our study, we found that increased TAD values were directly related to cut-out, which is in line with the reports of Geller et al. [15] and Lobo-Escolar et al. [16].

There is a consensus in the literature that lag screw positioning into the apex of the femoral head increases cut-out rates and leads to failure [16]. However, opinions differ as to whether the screw should be placed centrally [6,17,18] or tangent to the inferior part of the calcar [8,19,20]. In the present study, TAD <20 mm proved to be protective against the occurrence of failures, consistent with the reports of Pervez et al. [17]. Increased CalTAD values were more associated with failures and, therefore, represented an unfavorable place for inferior screw positioning.

**Figure 2:**
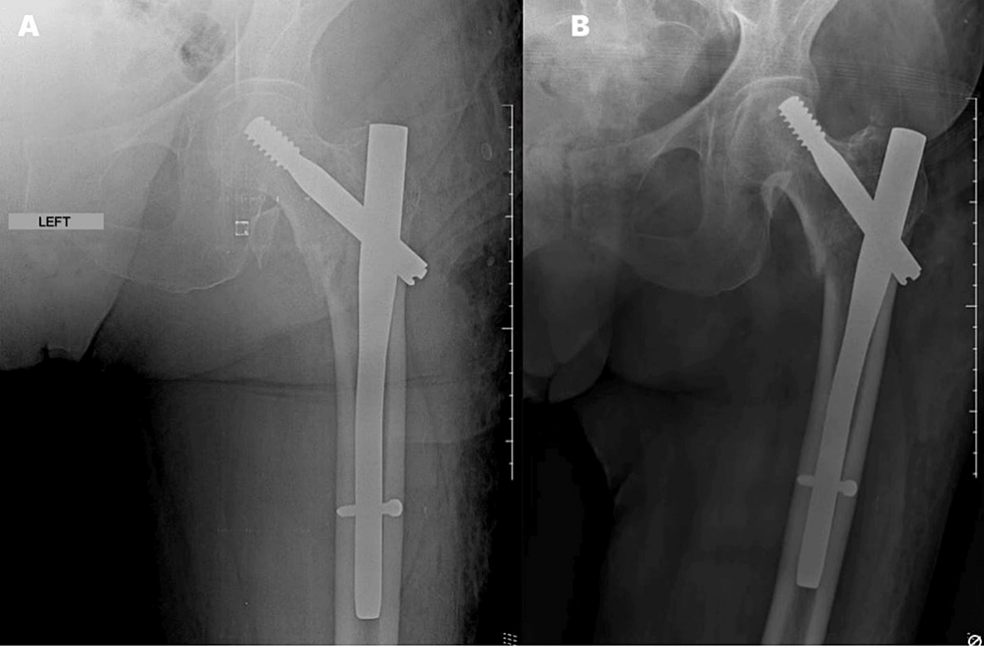
(A) Immediate postoperative radiograph and (B) after five weeks, showing cut-out.

**Figure 3:**
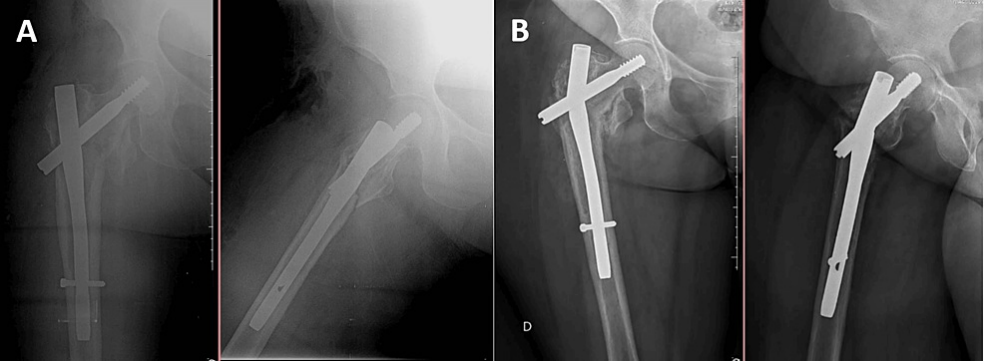
(A) Immediate postoperative radiograph and (B) after 12 weeks, showing implant breakage.

However, cut-out was not the main mode of nail failure observed in this study (n=4, 28.6%) alone (Figure 2), since implant breakage was slightly more common during the study period (n=5, 35.7%) (Figure 3). Implant breakages are considered rare events [1], being mainly associated with poor reduction quality (varus), use of short cephalomedullary nails for unstable fractures with subtrochanteric or reverse oblique extension, open reduction inducing devascularization or loss of the osteoinductive fracture hematoma, use of cerclage wires compromising periosteal vascularization, and poor surgical technique [21-23].

Varus reduction was the most important factor in nail failures in the present study (n=9, 19.1%; p<0.001), supporting the data reported in several previous studies [8,16,17]. We believe that the implant breakages reviewed in this study may also be attributed to this phenomenon, as varus reduction was observed in four (80%) of the five cases of implant breakage. The analysis of the other factors was found to be inconclusive, requiring further studies to evaluate this specific phenomenon.

**Figure 4:**
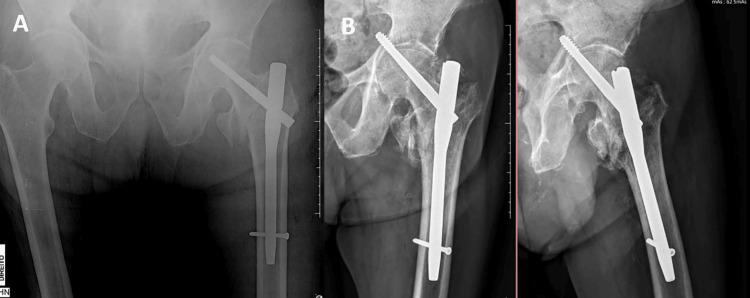
(A) Immediate postoperative radiograph and (B) after 13 weeks, showing cut-through.

Another complication was the occurrence of cut-through (n=3, 21.4%) (Figure 4), but its predisposing factors remain under debate [24,25]. Valgus reduction, iliotibial band forces, and severe osteoporosis may be related to the occurrence of this event, and the lateral migration and medial advancement of the blade cannot be ruled out [24]. The investigation of these events in our study did not find robust evidence of predisposing factors, proving inconclusive. Additional studies are needed to further elucidate the topic.

The overall failure rate in this study was 6.3% (n=13), with a 95% confidence interval. This rate is consistent with the findings of von Rüden et al. [1] (3%) and Kashigar et al. [8] (13%).

Limitations of this study include the retrospective design and small sample size. This may be explained by the large number of older people included in the study (mean age of 75 years) as well as the COVID-19 pandemic during the study period, which led to a high rate of patients not complying with the minimum outpatient follow-up of six months for inclusion in the study (n=96, 25.2%). Furthermore, the procedures were performed by multiple surgeons, with different levels of training and expertise, which may have influenced the postoperative results.

## Conclusions

In this study, failure of cephalomedullary nailing was observed in 13 (6.3%) cases, with implant breakage and cut-out being the most common events. The present article supports previous evidence that varus reduction potentially causes collapse and nail failure in pertrochanteric fractures treated with cephalomedullary nailing. It is also consistent with the findings that high TAD and CalTAD values contribute to the incidence of cut-out.

## References

[1] von Rüden C, Hungerer S, Augat P, Trapp O, Bühren V, Hierholzer C (2015). Breakage of cephalomedullary nailing in operative treatment of trochanteric and subtrochanteric femoral fractures. Arch Orthop Trauma Surg.

[2] Braithwaite RS, Col NF, Wong JB (2003). Estimating hip fracture morbidity, mortality and costs. J Am Geriatr Soc.

[3] Longo UG, Viganò M, de Girolamo L, Banfi G, Salvatore G, Denaro V (2022). Epidemiology and management of proximal femoral fractures in Italy between 2001 and 2016 in older adults: analysis of the National Discharge Registry. Int J Environ Res Public Health.

[4] Meinberg EG, Agel J, Roberts CS, Karam MD, Kellam JF (2018). Fracture and Dislocation Classification Compendium-2018. J Orthop Trauma.

[5] Johnson NA, Uzoigwe C, Venkatesan M, Burgula V, Kulkarni A, Davison JN, Ashford RU (2017). Risk factors for intramedullary nail breakage in proximal femoral fractures: a 10-year retrospective review. Ann R Coll Surg Engl.

[6] Baumgaertner MR, Curtin SL, Lindskog DM, Keggi JM (1995). The value of the tip-apex distance in predicting failure of fixation of peritrochanteric fractures of the hip. J Bone Joint Surg Am.

[7] Li S, Chang SM, Jin YM (2016). A mathematical simulation of the tip-apex distance and the calcar-referenced tip-apex distance for intertrochanteric fractures reduced with lag screws. Injury.

[8] Kashigar A, Vincent A, Gunton MJ, Backstein D, Safir O, Kuzyk PR (2014). Predictors of failure for cephalomedullary nailing of proximal femoral fractures. Bone Joint J.

[9] Lenich A, Vester H, Nerlich M, Mayr E, Stöckle U, Füchtmeier B (2010). Clinical comparison of the second and third generation of intramedullary devices for trochanteric fractures of the hip--blade vs screw. Injury.

[10] Bojan AJ, Beimel C, Speitling A, Taglang G, Ekholm C, Jönsson A (2010). 3066 consecutive gamma nails. 12 years experience at a single centre. BMC Musculoskelet Disord.

[11] Yoshino N, Watanabe Y, Takenaka N (2006). Implant failure of long gamma nail in a patient with intertrochanteric-subtrochanteric fracture. J Orthop Sci.

[12] Sadowski C, Lübbeke A, Saudan M, Riand N, Stern R, Hoffmeyer P (2002). Treatment of reverse oblique and transverse intertrochanteric fractures with use of an intramedullary nail or a 95 degrees screw-plate: a prospective, randomized study. J Bone Joint Surg Am.

[13] Baumgaertner MR, Curtin SL, Lindskog DM (1998). Intramedullary versus extramedullary fixation for the treatment of intertrochanteric hip fractures. Clin Orthop Relat Res.

[14] Park SR, Kang JS, Kim HS, Lee WH, Kim YH (1998). Treatment of intertrochanteric fracture with the gamma AP locking nail or by a compression hip screw--a randomised prospective trial. Int Orthop.

[15] Geller JA, Saifi C, Morrison TA, Macaulay W (2010). Tip-apex distance of intramedullary devices as a predictor of cut-out failure in the treatment of peritrochanteric elderly hip fractures. Int Orthop.

[16] Lobo-Escolar A, Joven E, Iglesias D, Herrera A (2010). Predictive factors for cutting-out in femoral intramedullary nailing. Injury.

[17] Pervez H, Parker MJ, Vowler S (2004). Prediction of fixation failure after sliding hip screw fixation. Injury.

[18] Larsson S, Friberg S, Hansson LI (1990). Trochanteric fractures. Influence of reduction and implant position on impaction and complications. Clin Orthop Relat Res.

[19] Parker MJ (1992). Cutting-out of the dynamic hip screw related to its position. J Bone Joint Surg Br.

[20] De Bruijn K, den Hartog D, Tuinebreijer W, Roukema G (2012). Reliability of predictors for screw cutout in intertrochanteric hip fractures. J Bone Joint Surg Am.

[21] Zafiropoulos G, Pratt DJ (1994). Fractured gamma nail. Injury.

[22] Wozasek GE, Radler C, Vécsei V (2002). Multiple gamma nail failure. Orthopedics.

[23] Gaebler C, Stanzl-Tschegg S, Tschegg EK, Kukla C, Menth-Chiari WA, Wozasek GE, Heinz T (1999). Implant failure of the gamma nail. Injury.

[24] Yam M, Kang BJ, Chawla A (2020). Cephalomedullary blade cut-ins: a poorly understood phenomenon. Arch Orthop Trauma Surg.

[25] Souza BG, Oliveira CD, Caires NV, de Sá LA, Cury GC (2021). Migração intrapélvica do parafuso cefálico de uma haste tipo gama: relato de um caso desafiador. HU Rev.

